# Social media distrust and turn to artificial intelligence among generation Z: a qualitative model linking digital minimalism and financial discipline with social policy recommendations

**DOI:** 10.3389/frai.2026.1684312

**Published:** 2026-03-19

**Authors:** Büşra Ökten

**Affiliations:** Istanbul Medeniyet Universitesi, Istanbul, Türkiye

**Keywords:** artificial intelligence, digital minimalism, financial discipline, Generation Z, social media trust, thematic analysis

## Abstract

**Purpose:**

This study examines how Generation Z navigates social media distrust and turns to artificial intelligence (AI) tools within the broader contexts of digital minimalism and financial discipline. By developing a qualitative thematic model, we aim to elucidate the motivations, behaviors, and interrelations among these phenomena and derive actionable social policy recommendations.

**Materials and methods:**

A semi-structured interview process was conducted with 27 Turkish Gen Z participants (ages 08–28), who were recruited via university mailing lists and social media groups. The interview guide combined demographic and socioeconomic items, fixed-response usage metrics (platform preferences, screen time), and open-ended prompts on experiences with misinformation, minimalist practices, budgeting behaviors, and AI adoption. Data were exported to NVivo 12 and analyzed using a six-phase thematic analysis framework (familiarization, coding, theme development, review, definition, and reporting). Inter-coder reliability (Cohen’s *κ* = 0.78) and member-checking procedures ensured analytic rigor.

**Findings:**

Four primary themes emerged: (1) pervasive skepticism toward social media information, with only 11.1% expressing high trust; (2) widespread adoption of digital minimalism practices by 59.3% of Participants to mitigate cognitive overload; (3) mixed engagement with formal budgeting tools, as only 37.0% employ systematic expense tracking despite high economic uncertainty; and (4) strong confidence in AI-generated recommendations by 70.4%, highlighting AI’s perceived impartiality and data-driven value. These domains intersect dynamically: distrust of social feeds often co-occurs with minimalist routines and AI reliance, while reduced screen time supports clearer financial decision making.

**Conclusion:**

Generation Z demonstrates a proactive stance toward reclaiming agency in digital and economic spheres by combining minimalism, selective use of financial-tech tools, and emerging AI platforms. To support balanced technology engagement and financial resilience, stakeholders should integrate media-literacy education, promote transparent algorithmic governance, and design accessible, ethically aligned AI-and fintech solutions.

## 1 Introduction

Generation Z, defined roughly as those born between 1997 and 2012, represents the first cohort to mature entirely within a digitally saturated environment. From uninterrupted access to smartphones in early adolescence to algorithm-driven newsfeeds in late teens, their formative experiences have been shaped by platforms engineered for engagement above all else (McKinsey and Company, 2024). Yet, as social media ecosystems have grown more complex—interweaving entertainment, news, commerce, and community—many young users report an increasing sense of alienation and fatigue (Pew Research Center, 2022). Digital interactions that once promised greater connectivity now often manifest as fragmented attention, anxiety about online image management, and a pervasive fear of missing out. Such dynamics have triggered critical reflection among Generation Z on the true costs of endless scrolling and the value of reclaiming unmediated, real-world experiences (Shahade and Deshmukh, 2025; Przybylski et al., 2013; Dhir et al., 2018).

Concurrently, rampant misinformation and manipulative content strategies have fostered widespread distrust in social media as a reliable source of truth (Frank and George, 2024). From sensationalized headlines to micro-targeted advertisements, the lines between factual reporting and persuasive messaging have blurred, leaving Gen Zers wary of the very platforms they use to stay informed. This skepticism extends beyond news consumption; it colors perceptions of peer-generated content, influencer endorsements, and even social conversations. For a generation accustomed to fact-checking via multiple browser tabs, the cognitive burden of verifying each piece of information can erode trust and diminish the perceived credibility of entire networks (Guess et al., 2020; De Veirman et al., 2017). As a result, many young adults increasingly question whether the benefits of social validation and instant updates outweigh the mental toll of navigating an environment rife with half-truths and hidden agendas (Maiti et al., 2025).

In response to these challenges, a counter-movement of digital minimalism has emerged, advocating for intentional and value-driven technology use (Newport, 2019). Rather than rejecting technology outright, digital minimalists emphasize clarity of purpose, pruning nonessential apps, and scheduling regular periods of “digital vacation” free from screens (Butt, 2024). These practices are designed to restore agency by shifting the locus of control back to the individual—prioritizing deep work, meaningful face-to-face interactions, and mindful leisure over dopamine-driven notifications (Syvertsen and Enli, 2019). Among Generation Z, early adopters of minimalist routines report improvements in concentration, reductions in anxiety, and a renewed appreciation for hobbies that predate the smartphone era (OECD, 2019). Such lifestyle adjustments serve as both a coping mechanism and a form of silent protest against the algorithmic architectures that commodify user attention (Dadwal et al., 2024).

Alongside evolving digital habits, Generation Z’s financial landscape has been marked by uncertainty: ballooning student debts, volatile gig economies, and global economic disruptions have amplified concerns about long-term stability (World Economic Forum, 2023). Against this backdrop, many Gen Zers seek out digital tools to enhance personal financial discipline (Mujtaba, 2025). Budget-tracking apps, automated saving algorithms, and peer-support communities enable users to visualize spending patterns, set savings milestones, and resist impulsive purchases fueled by influencer marketing (De Veirman et al., 2017). However, the same social media channels that drive cultural trends and aspirational lifestyles also expose young consumers to curated depictions of wealth and success—fostering a tension between financial prudence and the pressure to conform (Dittmar et al., 2014). Understanding how digital minimalism practices intersect with financial self-regulation is thus crucial for illuminating pathways to economic resilience in this generation (Schiff et al., 2021). In this study, financial self-regulation is understood not simply as a personal habit or a lifestyle choice, but as a practice that develops within everyday digital experiences and broader economic conditions.

Studies on Generation Z’s financial behavior indicate high vulnerability to impulsive digital consumption alongside increasing awareness of financial precarity, debt anxiety, and saving insecurity (OECD/INFE, 2023; Da Silva et al., 2023; Ghafoor et al., 2024). The coexistence of consumption pressure and financial risk sensitivity forms a central tension in Gen Z’s economic subjectivity. Interestingly, as trust in social media wanes and concerns about digital overload grow, artificial intelligence (AI) has surfaced as a promising alternative for information retrieval and decision support. Research on social media distrust and misinformation demonstrates that young users increasingly question algorithmic credibility, sponsored content transparency, and platform-driven manipulation (Vosoughi et al., 2018; Guess et al., 2020). This growing epistemic uncertainty reshapes Gen Z’s information-seeking strategies and accelerates their shift toward alternative verification channels.

Generation Z, having grown up alongside rapid advancements in machine learning, often views AI chatbots and recommendation systems as more consistent and data-driven than human-curated feeds (Glikson and Woolley, 2020). Whether soliciting budgeting advice, seeking unbiased news summaries, or exploring mental-health support, many young users appreciate AI’s capacity to distill complex inputs into actionable insights. At the same time, questions of algorithmic transparency, ethical data use, and potential biases in training datasets complicate the allure of AI as a panacea (Zhang and Dafoe, 2019; Logg et al., 2019). Examining how Generation Z negotiates these trade-offs—balancing AI’s analytical strengths against residual mistrust in digital intermediaries—can shed light on emerging paradigms of techno-cognitive empowerment (Nosike et al., 2024).

Despite a growing body of quantitative research on digital engagement metrics and consumer behavior, there remains a notable gap in qualitative frameworks that integrate social media distrust, digital minimalism, financial discipline, and AI orientation into a cohesive model. Most existing studies treat these phenomena in isolation, overlooking their interconnected nature within the lived experiences of young adults (Newport, 2019; Dhir et al., 2018; Glikson and Woolley, 2020). To address this, the present study draws on in-depth semi-structured interviews to construct a thematic model to construct a thematic model reflecting Generation Z’s strategies for navigating modern digital landscapes. By mapping the motivations, barriers, and adaptive practices that underlie technology use, the research offers nuanced insights into how digital minimalism and financial self-regulation co-evolve with AI adoption (Rosa et al., 2025).

Recent studies on digital minimalism emphasize intentional technology use as a protective strategy against cognitive overload, emotional exhaustion, and fragmented attention (Newport, 2019). Digital well-being literature further highlights that excessive platform exposure among Gen Z is associated with increased anxiety, sleep disruption, and reduced self-regulation capacities (Odgers and Jensen, 2020; Twenge and Campbell, 2019). Recognizing that individual choices are nested within broader socio-technical systems, this study proposes targeted social policy recommendations aimed at fostering healthier digital environments. These include educational initiatives to enhance media literacy, regulatory measures to increase algorithmic transparency, and support for community-based programs that promote mindful technology practices. By aligning policy interventions with the articulated needs of Generation Z, stakeholders can help to cultivate digital ecosystems that prioritize well-being, trust, and economic empowerment—ultimately guiding young adults toward more balanced and sustainable relationships with technology (Shifa et al., 2025).

However, despite this expanding literature, these domains—digital minimalism, social media distrust, financial discipline, and trust in AI—are still predominantly examined in isolation. There remains a significant lack of integrative qualitative models that empirically explore how these dimensions structurally interact within the lived experiences of Generation Z. This study positions artificial intelligence not merely as a comparative reference to social media but as a central analytical lens. The proposed qualitative model conceptualizes AI not as a primary source of epistemic trust, but as a compensatory mechanism that interacts with digital minimalism and financial discipline. Specifically, AI is examined as a pragmatic resource that Gen Z turns to when traditional digital ecosystems become cognitively overwhelming or informationally unreliable (Zhang and Dafoe, 2019; Pachegowda, 2023).

## 2 Materials and methods

### 2.1 Study design and population

#### 2.1.1 Study design and participants

This research employed a qualitative descriptive design to capture Generation Z’s experiences and practices. Participants were 27 self-identified Gen Z individuals (aged 18–28) residing across various cities in Türkiye. Recruitment took place via convenience sampling: invitations to participate in the study were shared through university mailing lists and social media groups frequented by Gen Z. Inclusion criteria required (a) birth year between 1997 and 2007, (b) regular use of at least one social media platform, and (c) fluency in Turkish or English. A total of 28 people participated in the interview; however, one participant was excluded from the analysis because they did not meet the age criterion. The final sample consists of 27 people. The sample included 15 female and 12 male participants, with diversity in educational background and self-reported socio-economic conditions. While not statistically representative, this composition supports the qualitative aim of capturing varied digital experiences among Turkish Generation Z youth.

This qualitative descriptive design was selected because the study aims to explore Gen Z’s lived experiences and subjective reasoning patterns related to digital minimalism, financial discipline, and engagement with AI, phenomena that are not easily captured through quantitative measures.

Each construct in the study was operationalized through targeted thematic prompts in the semi-structured interview guide.

- Digital minimalism was explored through questions on device-limiting strategies, notification control, app removal, and intentional use.
- Financial discipline was assessed using prompts about budgeting habits, impulsive spending triggers, and emotional responses to financial decisions.
- Social media distrust was examined through questions on misinformation encounters, verification strategies, and perceived platform reliability.
- Through questions about the perceived accuracy of AI-generated information, expectations regarding AI-assisted decision-making, and participants’ reported usage patterns of AI tools, the study investigated whether perceptions of trust in AI are limited and context-dependent.

#### 2.1.2 Interview procedure

Data were gathered through semi-structured, one-on-one interviews conducted either face-to-face or online by the researcher. No audio recordings were made; instead, detailed field notes were taken during each interview. At the end of each session, participants were asked to review and confirm the notes for accuracy. Interviews were guided by a thematic framework covering the following four areas:

- Demographics & socioeconomics: age, gender, education level, city of residence, and monthly personal income.
- Digital usage & trust: preferred platforms, average daily screen time, experiences with misinformation, and strategies for verifying online content.
- Digital Minimalism & financial behavior: habits for limiting device use (e.g., notification management, app pruning), budgeting practices, and emotional responses to impulsive spending.
- AI Engagement with AI tools: frequency and context of AI-tool use (e.g., chatbots, recommendation systems), trust in AI versus social media, and perceived strengths or limitations of AI in decision-making.

Open-ended prompts invited narrative detail, while fixed-response items captured key demographics. A pilot test with three Gen Z volunteers refined wording for clarity and ensured cultural relevance. The structure of the interview guide directly reflects the study’s research questions. Each thematic block (digital usage, minimalism, financial discipline, and AI-related perceptions, including limited and context-dependent trust considerations) was designed to generate qualitative evidence relevant to the proposed conceptual link between digital behavior, financial practices, and AI-mediated decision-making. Although AI is treated as a broad analytical category, participants’ accounts centered predominantly on ChatGPT, with only limited reference to other platforms. Technical distinctions between tools were rarely emphasized; instead, AI was described mainly as an accessible, conversational decision-support resource. Accordingly, AI tools are analyzed collectively, while acknowledging that platform-specific affordances may shape trust perceptions differently.

#### 2.1.3 Data collection procedure

Interviews were conducted between 5 July and 20 July 2025. Prior to each session, participants received an information sheet and provided verbal informed consent. All interviews were carried out by the researcher, either face-to-face or via online video platforms. Instead of audio recording, detailed notes were taken during each interview session. At the end of each interview, participants were invited to review and verify the notes to ensure accuracy. No personally identifying information was collected beyond general demographics. To strengthen methodological transparency and replicability, the full semi-structured interview protocol is now provided in the Appendix. This study is situated in the Turkish context, where high social media use, rapid digital transformation, and evolving economic conditions shape Generation Z’s everyday digital practices, trust perceptions, and self-regulation strategies.

### 2.2 Data analysis

All interview notes were imported into NVivo 12 for thematic analysis, following a six-phase process:

- Familiarization: repeated reading of all responses to gain holistic insight.
- Initial coding: segmentation of meaningful excerpts and assignment of descriptive codes.
- Theme development: grouping related codes into candidate themes reflecting core patterns.
- Review & refinement: checking each theme for internal consistency and distinctiveness.
- Definition & naming: articulating clear definitions and labels for final themes.
- Reporting: illustrative participant statements (as paraphrased in contemporaneous field notes) were selected to support each theme and the findings were structured in direct alignment with the research aims. For reliability purposes, the coding framework was independently reviewed by a second qualitative researcher who was not involved in the authorship of the manuscript. Inter-coder agreement (Cohen’s *κ* > 0.75) was calculated on a randomly selected subset of the data. Discrepancies were resolved through structured consensus discussions, and the finalized codebook was then applied to the full dataset. This detailed description of the coding and analysis process is provided to enhance analytic transparency and allow for methodological replication in future studies.

### 2.3 Trustworthiness and validation

To bolster credibility and dependability, the study incorporated:

- Analytic cross-validation: themes were compared across participant subgroups such as gender and income level.
- Member checking: sharing thematic summaries with five participants for feedback and clarification.
- Reflexivity: maintaining analytical memos to surface and bracket researcher assumptions.
- Audit trail: documenting all methodological decisions and codebook revisions. Together, these procedures were implemented to increase the credibility, dependability, and replicability of the qualitative findings

### 2.4 Ethical considerations

Ethical approval was obtained from the Institutional Review Board of [Istanbul Medeniyet Üniversitesi] (Protocol No: E-38510686-050.04-2500042461). Participation in the study was voluntary, anonymous, and could be withdrawn at any stage without consequence. All research procedures adhered to the ethical principles outlined in the Helsinki Declaration and national regulations. Interview notes were securely stored in encrypted digital files, accessible only to the principal researcher.

## 3 Findings

The thematic analysis of interview data from 27 Generation Z participants revealed a nuanced and multifaceted portrait of how young adults navigate the intersections of digital trust, intentional technology use, economic self-management, and emerging AI engagement. Across the dataset, four overarching themes emerged: first, a pervasive skepticism toward social media as a credible information source, driven by frequent exposure to misinformation and algorithmic manipulation; second, the deliberate adoption of digital minimalism practices—ranging from app pruning and notification silencing to structured “digital morning” routines —as a means of reclaiming attention and reducing cognitive overload; third, financially prudent behaviors encompassing budget tracking, automated saving tools, and conscious resistance to impulsive, influencer-driven spending; and fourth, an increasing reliance on AI-powered interfaces for unbiased insights, decision support, and streamlined information retrieval. Crucially, these themes do not exist in isolation but interweave in participants lived experiences: distrust of social feeds often catalyzed both minimalism efforts and the turn to AI, while financial discipline practices were reinforced by AI-driven budgeting aids and the mental clarity afforded by reduced screen time. These relationships should be interpreted as perceived, context-dependent, and mutually reinforcing dynamics rather than linear causal sequences, consistent with modeling studies showing that information adoption and trust shifts emerge from interacting cognitive, emotional, and contextual factors rather than simple cause–effect mechanisms (Zhang et al., 2025). In the sections that follow, we unpack each thematic domain in detail, illustrating the dynamic strategies Generation Z employs to balance well-being, economic stability, and digital engagement in an era of unprecedented technological complexity.

Table 1 gender distribution reveals a modest female majority (55.6%) alongside a substantial male representation (44.4%), ensuring that insights drawn from subsequent thematic analysis reflect the perspectives of both young women and men. This balance is important given documented gendered nuances in digital behavior.

**Table 1:** Gender distribution.

Gender	*n*	%
Female	15	55.6%
Male	12	44.4%
Total	27	100%

The birth-year breakdown in Table 2 underscores a strong presence of the youngest Gen Z cohort: nearly one-third of Participants were born in 2007, while only isolated individuals hail from the late-1990s end. The largest share of participants was born in 2007, representing 33.3% of the sample. The percentage distribution of the remaining age groups ranges between 3.7 and 11.1%. This concentration among 18-year-olds suggests that early-career and teenage voices dominate the dataset, reflecting acute attitudes toward both schooling pressures and nascent financial independence.

**Table 2:** Birth year distribution.

Birth year	*n*	%
1997	1	3.7%
1998	1	3.7%
1999	1	3.7%
2000	2	7.4%
2001	2	7.4%
2003	2	7.4%
2004	3	11.1%
2005	2	7.4%
2006	4	14.8%
2007	9	33.3%
Total	27	%100

Education levels (Table 3) span high school (37.0%), university (55.6%), and master’s studies (7.4%), indicating that most participants are still in formal education.

**Table 3:** Education level.

Education	*n*	%
High school	10	37.0%
University	15	55.6%
Master’s	2	7.4%
Total	27	100%

With 77.8% of participants identified as full-time students and the remaining evenly split between part- and full-time employed (Table 4).

**Table 4:** Employment status.

Status	*n*	%
Student	21	77.8%
Part time employed	3	11.1%
Fulltime employed	3	11.1%
Total	27	100%

**Figure 1:**
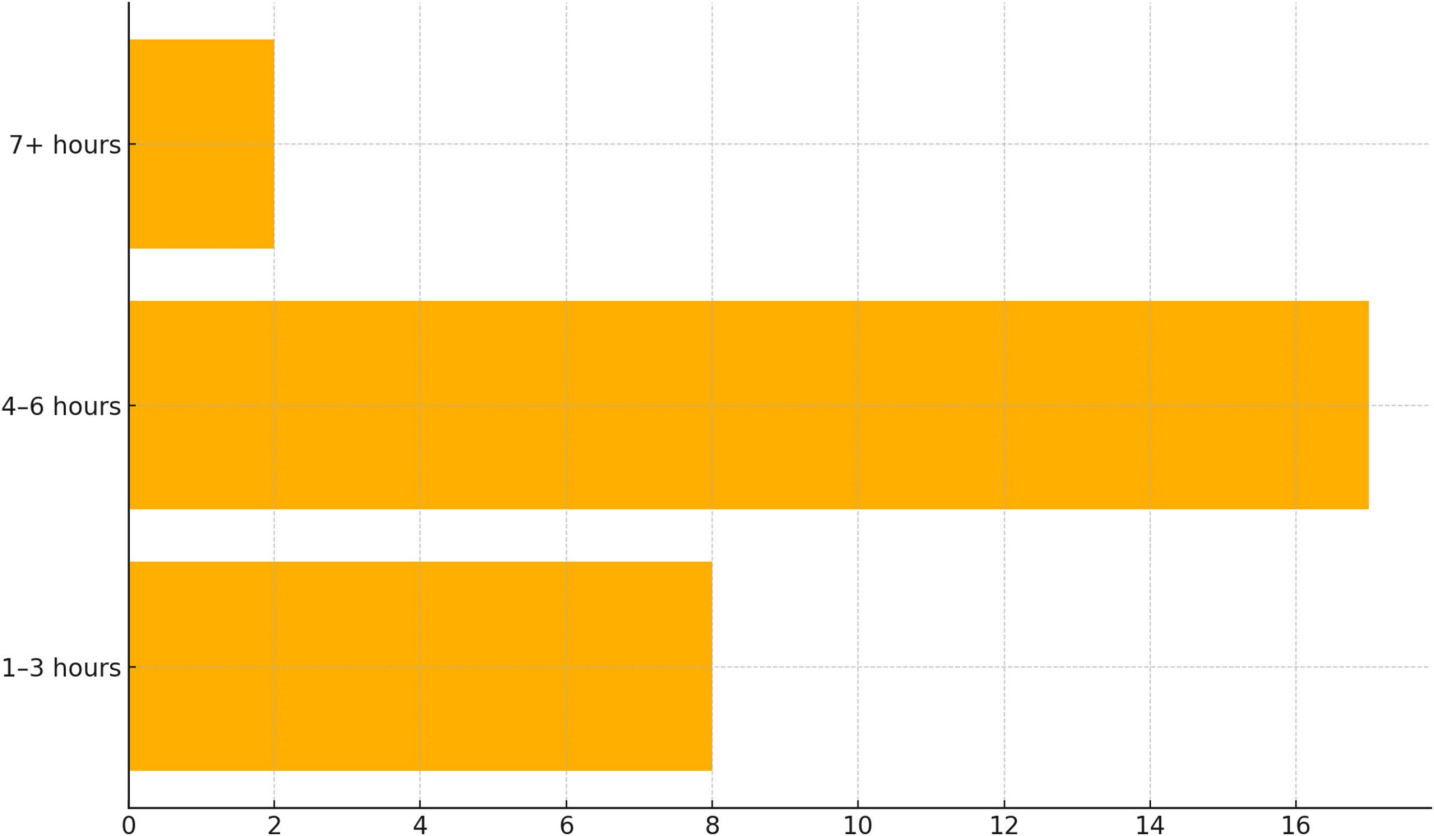
Daily screen time categories.

Figure 1 displays daily screen-time categories using a horizontal bar chart. The dominant category, 4–6 h per day, accounts for 63% of participants, highlighting a substantial baseline of digital engagement that extends well beyond leisure into study and work. Comparatively, only 7.4% exceed 7 h. The high level of daily screen exposure was also strongly reflected in the participants’ narratives. For example, one participant described their digital intensity as follows:

‘My phone is always within reach, and even when I don’t actually need to check it, I’m constantly on my phone out of sheer habit. I think my usage is largely unnecessary.’ (K11, age 19).

Over half of participants (59.3%) report adopting at least one digital minimalism practice (Table 5), such as disabling notifications, uninstalling nonessential apps, or instituting device-free periods. The drop from 16 adopters to 11 non-adopters illustrates that a clear majority (59.3%) have embraced at least one minimalism practice. This differential, while not vast, indicates that more participants reported actively attempting to reclaim attention from algorithm-driven platforms than those who reported no change in their digital practices. A participant who stated that they actively use digital minimalism practices in their life expressed the following:

**Table 5:** Adoption of digital minimalism practices.

Practices adopted?	*n*	%
Yes	16	59.3%
No	11	40.7%
Total	27	100%

“I’ve simplified my social media platforms. I also regularly monitor my screen time on my phone and try to keep it at an average level. Even for WhatsApp, many of my groups are archived, and some are on silent mode”. (K9, Age 24)

Despite growing awareness of financial precarity, only 37.0% of Participants utilize formal budgeting or expense-tracking systems (Table 6). This difference indicates that while many Gen Zers recognize the importance of financial discipline, consistent implementation varies across participants. Those who track expenses report feeling empowered by real-time insights into spending patterns and the ability to set and monitor savings goals often describing these practices in relation to digital tools they use. In contrast, the majority who do not use such systems often cite reasons like perceived complexity, lack of time to maintain records, or insufficient disposable income to warrant formal budgeting. This pattern is echoed in participants’ own accounts. One participant described their approach as follows:

**Table 6:** Use of budgeting or financial tracking systems.

System used?	*n*	%
Yes	10	37.0%
No	17	63.0%
Total	27	100%

“I don’t follow a specific financial plan; I just try to stay away from unnecessary expenses.” (K15, Age18).

**Figure 2:**
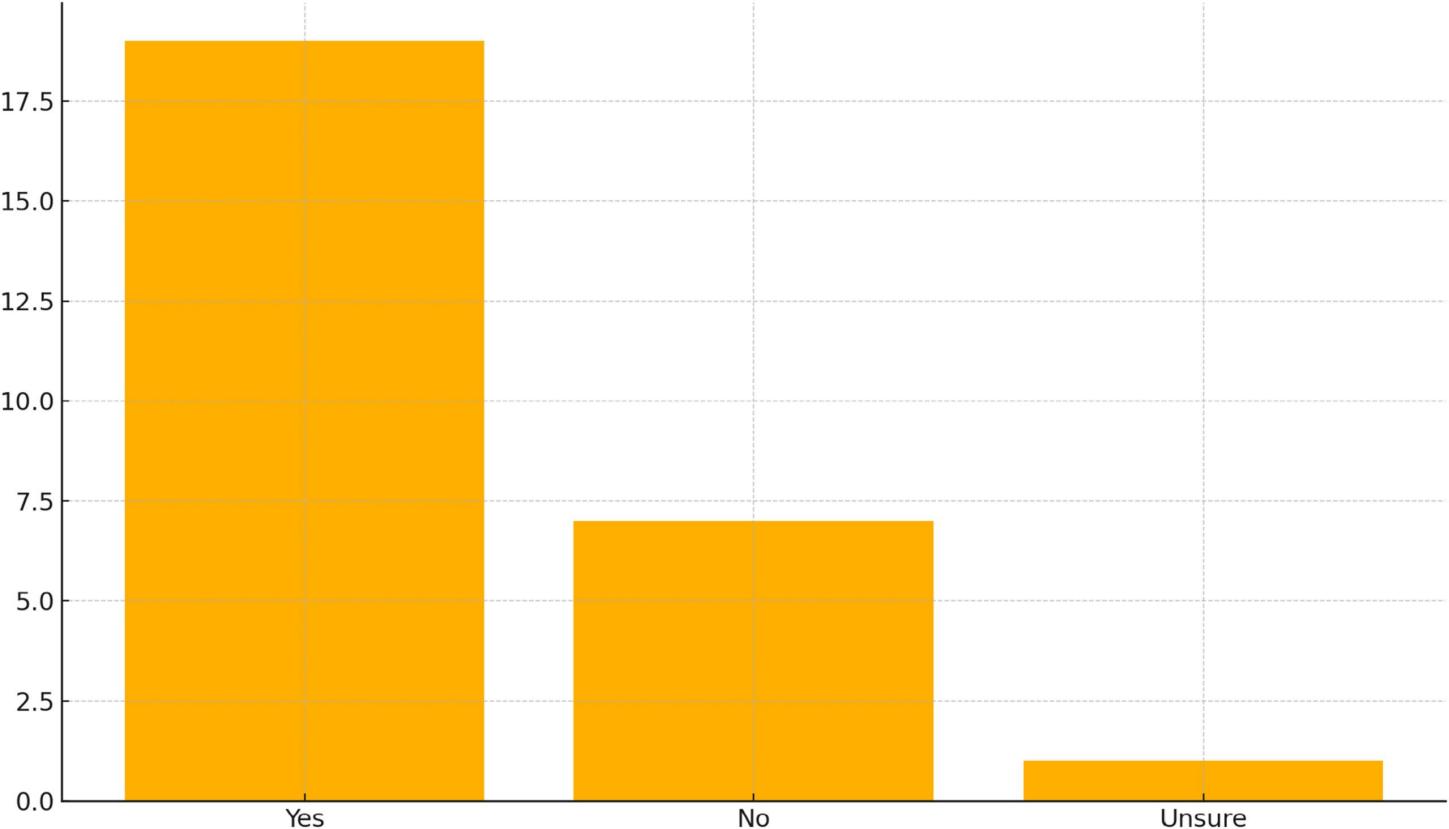
Trust in AI-generated recommendations.

Figure 2 illustrates participants’ self-reported trust in AI-generated recommendations. A high bar at “Yes” (19 Participants) contrasts sharply with “No” (7) and “Unsure” (1). Rather than indicating deep or unconditional epistemic trust, this pattern reflects Generation Z’s comparative confidence in AI systems when evaluated alongside social media feeds (see Table 7).

**Table 7:** Trust in social media information.

Level of trust	*n*	%
High trust	3	11.1%
Partial trust	16	59.3%
Low trust	8	29.6%
Total	27	100%

Within the qualitative model, AI trust is interpreted as a context-dependent and pragmatic perception, shaped by experiences of digital fatigue and informational overload. In the interview protocol, AI-related items were designed to capture participants’ cognitive, emotional, and behavioral reasoning underlying shifts from social media toward AI-supported decision-making. Participants’ accounts suggest that trust judgments often draw on perceived speed, accessibility, and informational capacity rather than assumptions of absolute accuracy. As one participant stated:

“I trust it because it can access most of the universal knowledge that is available within seconds.” (K7, age 20).

Trust in social media information is decidedly cautious: only 11.1% express high trust, with the majority (59.3%) indicating partial trust and a notable 29.6% reporting low trust (Table 8). One participant also expressed distrust in social media information, stating:

**Table 8:** Trust in AI generated recommendations.

Trust level	*n*	%
Yes	19	70.4%
No	7	25.9%
Unsure	1	3.7%
Total	27	100%

“I think the accuracy rate is low. It’s hard to get accurate information because people spread misinformation for engagement”. (K25-18)

A robust majority (70.4%) voice confidence in AI-generated recommendations, contrasting with their guarded stance toward social media (Table 8). One participant explains why they trust artificial intelligence on this issue but not social media:

“It’s very difficult to access accurate information. Artificial intelligence like GPT does a good job in this regard” (K2, Age 19).

Participants cite AI’s perceived impartiality, data-driven rationale, and ability to synthesize large information volumes as key strengths—qualities highly valued when making financial or life-planning decisions. However, the 25.9% who distrust AI often reference concerns about hidden biases in training data, opaque algorithmic logic, and data-privacy implications. The small “unsure” group reflects ambivalence rooted in limited firsthand experience with advanced AI tools.

### 3.1 Supplementary descriptive associations

**Figure 3:**
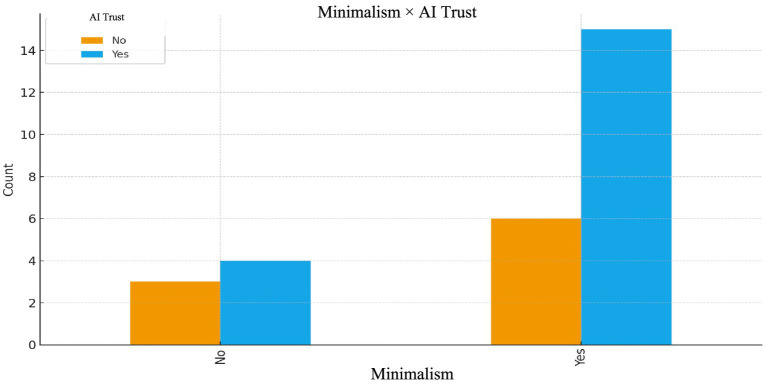
Minimalism × AI trust.

In addition to the thematic findings, several descriptive cross-tab analyses were conducted to visualize the relational patterns observed in the dataset. Although the study is qualitative in nature, these supplementary graphics help contextualize the themes by illustrating how digital minimalism, screen-time intensity, financial-tracking habits, and self-reported confidence in AI co-occur at the behavioral level (see Figure 3).

The cross-tabulation indicates that participants who practice digital minimalism tend to express higher levels of self-reported trust in AI-generated recommendations. As minimalist individuals intentionally reduce social media exposure due to concerns about misinformation and cognitive overload, AI tools are perceived as a more neutral, structured, and information-driven alternative.

**Figure 4:**
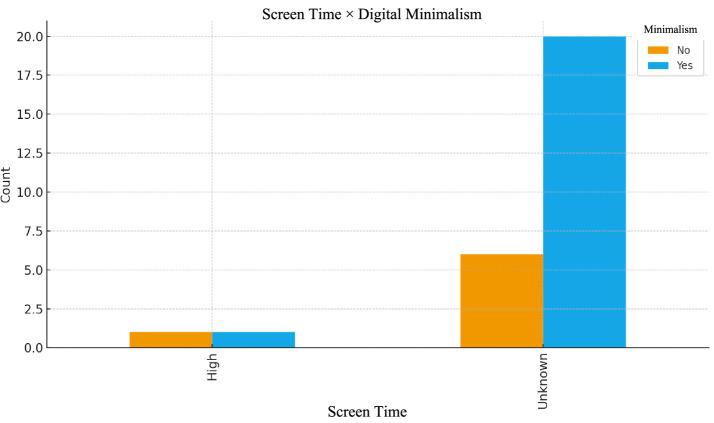
Screen time × digital minimalism.

Across the dataset, AI trust is better understood as a context-dependent and pragmatic component of participants’ decision-making processes, rather than as a standalone or unconditional epistemic stance. Participants described AI as a stabilizing mechanism that helps manage digital overload and informational uncertainty, positioning AI trust as an important—but not exclusive—dimension within the proposed model. In response to the question about exploring digital minimalism:

“I tried reducing my usage and succeeded. When I set and implemented daily goals and tasks for myself outside of digital usage, I successfully reduced my usage. At the end of the day, I feel like I’ve finished the day with a clearer mind.” In response to the question about trust in artificial intelligence, one participant stated:

“When I use it in scientific research, it has a 95% accuracy rate, and I can trust it in that regard” (K27, Age 22) (see Figure 4).

The relationship between daily screen time and digital minimalism shows that participants with higher screen exposure (≥4 h) are more likely to engage in minimalist strategies. Elevated screen time appears to accelerate cognitive fatigue, prompting behaviors such as disabling notifications, limiting app use, and implementing device-free intervals. A participant who used screens for 1–3 h per day stated that they felt mentally relaxed during a period when they reduced their screen time and practiced digital minimalism:

“I didn’t notice any financial difference, but I consciously distanced myself from social media. I closed my accounts earlier. I felt very relaxed mentally. My melancholy decreased significantly. I was a happy person.” (K26-Age 24).

**Figure 5:**
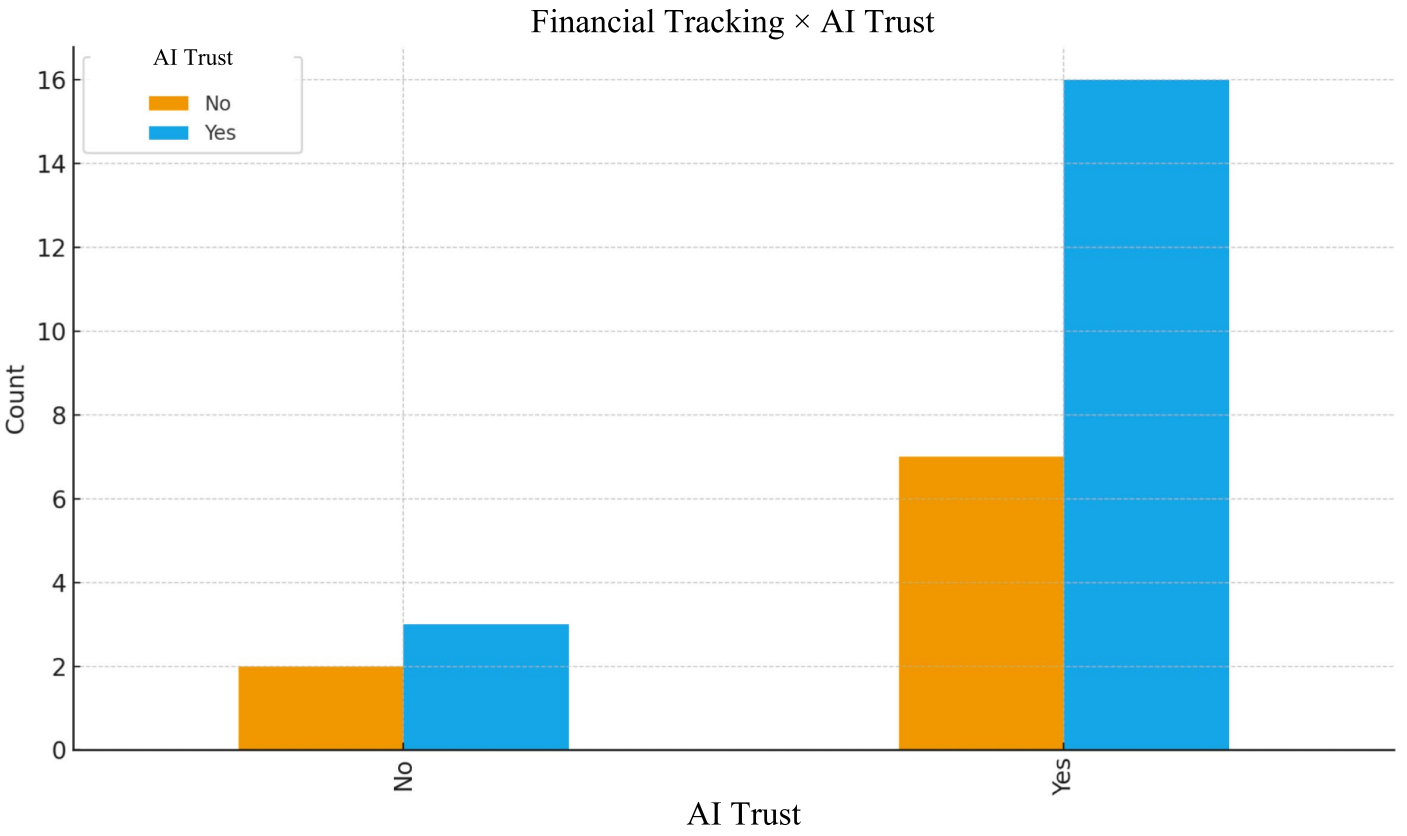
Financial tracking × AI trust.

Participants who actively use financial-tracking systems tend to report higher levels of self-reported trust in AI-supported recommendations. This association may reflect that financially disciplined individuals interpret AI tools as structured and rational decision-support systems, particularly for budgeting, spending analysis, and long-term planning. A participant who stated that they do not use financial tracking applications expressed the following regarding trust in artificial intelligence:

“I don’t trust it too much. I am the one who knows my situation best, and I don’t think I can explain it well to GPT in detail” (K24- Age 19) (see Figure 5).

## 4 Discussion

The demographic profile of our sample—predominantly female (55.6%) and heavily weighted toward the youngest Gen Z birth-years (29.6% born in 2007)—provides critical context for interpreting the patterns of digital engagement and coping strategies uncovered in this study. Overall, participants’ engagement with AI in this study is characterized more by pragmatic reliance than by deep epistemic trust, while AI orientation frames the broader attitudinal context within which these practices emerge. Although the literature on trust in artificial intelligence has grown significantly, most studies treat trust in AI as an individual-level psychological judgment focused on accuracy, reliability, or risk perceptions, and largely rely on experimental or quantitative designs (Baloğlu et al., 2018). Recent experimental work also indicates that AI trust and engagement are shaped by interaction design, with user-initiated assistance supporting greater agency than unsolicited guidance (Gressel et al., 2025).

This study contributes to the field by focusing on perceptions of trust in AI within a broader socio-digital context shaped by social media distrust, digital minimalism, and financial self-regulation, rather than treating trust in AI as an independent construct. From this perspective, trust in AI emerges not as a stable or purely technological judgment, but as a context-dependent and compensatory orientation that develops alongside daily strategies for managing cognitive overload and information uncertainty. As a qualitative and cross-sectional study, this research does not seek to account for every temporal or functional factor shaping digital minimalism, financial discipline, or AI-related practices. Even so, participants’ narratives suggest that these practices often unfold through gradual shifts—from intensive social media use to fatigue, selective withdrawal, and, in some cases, a growing reliance on AI tools. Rather than fixed patterns, these accounts point to adaptive processes that evolve over time, echoing broader research on the changing cognitive effects of digital technologies and artificial intelligence (Zhang et al., 2023).

Where prior research suggests females may experience higher social media–related anxiety and males may engage differently with gaming and news feeds (Baloğlu et al., 2018). In our sample, the slight female predominance may amplify reports of digital fatigue and mistrust, yet the near-equitable split provides confidence that observed patterns—such as adoption of digital minimalism or orientation toward AI tools—are not unduly driven by one gender’s unique experiences (Twenge and Martin, 2020). Future analyses may probe whether female participants more readily embrace notification silencing or whether male participants exhibit distinct approaches to financial tracking, but the current balance supports a broadly applicable qualitative model.

As mostly full-time students (77.8%) navigating high-school and university demands, these young adults report an average daily screen time of 4 to 6 h (63.0%), a level that, while necessary for academic and social functions, appears to precipitate significant cognitive and emotional strain (Thomée, 2018). Such a skew likely intensifies the emphasis on digital minimalism—as these younger Participants often juggle academic screen time with social media leisure—and shapes perceptions of AI as a study aid or homework support (Holmes et al., 2019). Conversely, the fewer participants from older birth years may offer glimpses into transitions toward full-time employment and longer-term budgeting, but their smaller number constrains generalization. Recognizing this distributional nuance is vital when interpreting themes around digital trust, as the youngest participants’ formative experiences differ from those of early Millennials who first encountered social media in adulthood. High-school and early-university perspectives dominate our findings, and any age-related patterns—like digital minimalism or AI usage—must be considered against this youthful baseline (Anderson and Jiang, 2018).

The fact that nearly 60% of participants have adopted at least one digital minimalism practice—whether app pruning, notification silencing, or scheduled device-free windows—underscores a collective recognition that unrestrained connectivity undermines focus, well-being, and study efficacy (Lund et al., 2024). The visual emphasis on the university segment underscores the academic pressures and hybrid learning contexts many Participants face—factors likely driving both their digital fatigue and exploration of productivity-enhancing AI tools. University students in particular bring firsthand insight into the pressures of online learning environments, hybrid classrooms, and academic screen-time requirements—factors that can exacerbate digital fatigue and fuel minimalism strategies outside class hours (Dhir et al., 2018). High-school Participants, on the other hand, may view social media through a peer-comparison lens that heightens FOMO, contributing to both distrust of sensationalized content and a desire to restrict exposure (Przybylski et al., 2013). The small postgraduate contingent likely balances academic reading, research-related AI tool use, and budgeting for living expenses, offering a window into how advanced learners integrate AI for literature summarization or automated citation assistance (Kasneci et al., 2023). Overall, the predominance of learners suggests that educational context heavily mediates Gen Z’s enactment of digital minimalism and financial discipline. At the same time, the 40.7% who have yet to implement such practices may lack clear models for intentional disengagement or fear social exclusion if they reduce their online presence.

The dataset reflects Generation Z’s transitional life stage—where academic commitments often outnumber sustained work obligations (Arnett, 2000). Students juggling assignments and exam preparation may exhibit stronger motivations to prune social media usage, viewing digital minimalism as a tactic to protect study zones and concentrate on deadlines (Reinecke and Hofmann, 2016). In contrast, the employed minority must negotiate workplace demands—remote or in-office—that shape their screen-time patterns and potentially their receptivity to AI-driven productivity tools (Brynjolfsson et al., 2023). Part-time workers, who straddle both spheres, might uniquely leverage AI for time management or budgeting apps for irregular incomes. This diversity of status illuminates how role demands interact with digital behaviors, emphasizing the need for tailored social policies that consider students’ and workers’ distinct challenges.

While the findings of this study are firmly grounded in the Turkish digital context, briefly engaging with qualitative research from other Global South settings helps clarify their situated character. For instance, studies on adolescents’ and young adults’ participation in TikTok challenges in South India point to the importance of peer affirmation, emotional reward, and platform-specific norms in shaping everyday digital practices (Roth et al., 2021). By contrast, the accounts of Turkish Generation Z participants in this study suggest that growing distrust in social media and experiences of cognitive fatigue play a more central role in motivating withdrawal from platforms and the search for alternative digital strategies. This observation is not intended as a direct comparison, but rather as a way of situating the findings within a broader socio-cultural landscape and highlighting their international relevance. Future research could extend this perspective through comparative cross-cultural qualitative studies, longitudinal designs that trace shifts in digital trust over time, or quantitative testing of the proposed model in different national contexts.

Educational institutions face an opportunity to integrate minimalism principles into curricula equipping students with practical tactics to manage screen-time demands without compromising academic collaboration or peer communication.

Spending nearly half the waking day online situates most Participants in a moderate-to-high usage bracket—enough to cultivate both digital proficiency and exposure to misinformation. Those in the 4–6 h range face a dual reality: their engagement is essential for academic, social, and entertainment needs, yet it poses a risk of cognitive overload and disrupted routines (Misra and Stokols, 2012). The lighter-use subgroup may represent individuals who already prioritize minimal digital footprints or whose work and study contexts limit screen dependence (Vanden Abeele, 2020). The heavy-use minority signals potential vulnerability to addictive engagement loops and algorithmic targeting, underscoring the importance of minimalism interventions for that group. Together, these patterns highlight the nuanced interplay between necessary digital immersion and the desire for intentional disengagement.

Such interventions could normalize digital sabbath routines and establish community-driven challenges, fostering a system-wide culture of mindful technology use rather than framing minimalism as an individual burden (Nadella et al., 2025; Sheikh, 2020). This situation uptake suggests that Generation Z is actively seeking strategies to reclaim focus and mental well-being in the face of pervasive online stimuli. Participants who implemented these measures frequently described enhanced productivity, reduced anxiety, and a clearer boundary between online and offline life—outcomes that align with behavioral theories on habit formation and attention restoration. Meanwhile, the 40.7% who have not adopted such practices may either underestimate the personal costs of unchecked usage or lack accessible models for disciplined disengagement. Understanding barriers—such as fear of social exclusion or perceived necessity of instant connectivity—will be crucial for designing educational interventions that encourage minimalism without stigmatizing digital participation.

Parallel to efforts at self-regulation, pervasive skepticism toward social media information—and the limited reliance on formal fact-checking mechanisms—emerge as defining features of Generation Z’s digital landscape (Abdumannopova, 2025; Pragith et al., 2024). Although only 11.1% of Participants express high trust in social feeds, most (59.3%) maintain partial confidence, engaging in reactive verification behaviors such as cross-referencing multiple sources, and nearly 30% maintain overt distrust. This guarded stance appears to catalyze not only minimalism behaviors but also a strategic pivot toward alternative knowledge sources. Ironically, despite their wariness of algorithm-driven content, many Gen Zers continue to rely on the immediacy and communal cues of social platforms for socialization and trend tracking, creating a tension between reliance and suspicion (Pandey, 2025). Addressing this ambivalence will require multi-layered media-literacy initiatives that move beyond generic warnings about “fake news” to teach heuristics for assessing content provenance, understanding platform incentives, and leveraging community fact-checking networks. Moreover, social media companies must be held accountable for greater transparency in how recommendation algorithms prioritize content, enabling users to make informed choices rather than surrendering control to opaque engagement engines (Ali et al., 2023).

Low level of trust in social media reflects acute awareness of misinformation risks and algorithmic persuasion tactics. Partial-trust Participants often engage in fact-checking behaviors—cross-referencing multiple sources or consulting authoritative news outlets—yet they still rely on social channels for immediacy and community sentiment. The low-trust segment largely eschews social media for critical information, preferring traditional media or direct expert consultations. These attitudes highlight the urgent need for media-literacy curricula that equip Gen Z with evaluative frameworks for assessing online content and for platform-level reforms to enhance transparency around source provenance and content labeling. These findings demonstrate that the methodological choices allowed the study to adequately capture the behavioral, emotional, and cognitive mechanisms underlying Gen Z’s interaction with digital media and AI systems (Vosoughi et al., 2018; Guess et al., 2020). Distrust of social media and cognitive load stand out as the main contextual factors shaping participants’ orientation toward AI-powered tools; however, the findings also show that this orientation cannot be explained solely by these factors. Additional effects can also be observed, pointing to assumptions attributed to the perceived academic benefit of AI tools and the novelty effect within everyday decision-making practices, which enable a more comprehensive assessment of perceptions of trust in AI.

Our findings also indicate that those who do employ budgeting tools report heightened confidence in their ability to allocate funds, resist impulse purchases, and sustain savings goals. These findings underscore an opportunity for fintech designers and educators to simplify financial-tracking tools and integrate them seamlessly with platforms Gen Z already uses, thereby lowering activation energy for disciplined money management (Widyaningrum et al., 2024).

Against this backdrop of digital skepticism and minimalism, financial discipline practices reveal a notable implementation gap. Although this research Gen Z expresses acute awareness of economic precarity—underscored by the 37.0% who use formal budgeting or expense-tracking systems—they are outnumbered by the 63.0% who do not. This disparity suggests that recognition of the importance of financial self-management does not automatically translate into systematic habits. Indeed, many non-users cite perceived complexity, time constraints, or insufficient disposable income as barriers to consistent financial tracking. Yet our data also indicate that those who do employ budgeting tools report heightened confidence in their ability to allocate funds, resist impulse purchases, and sustain savings goals (Widyaningrum et al., 2024). Given the prevalence of influencer marketing and in-app shopping prompts within social media, financial-tech developers and educators must co-design lightweight, intuitive budgeting interfaces—potentially embedded within platforms Gen Z already frequents—to lower the activation threshold for disciplined spending (Lou and Yuan, 2019). School-based financial literacy modules could further reinforce these tools by demonstrating real-world applications, from splitting rent to planning for emergent expenses, thereby contextualizing budgeting as an empowering skill rather than an onerous chore (Ali et al., 2023).

Enthusiasm for AI stems from perceptions of algorithmic impartiality, data-driven rigor, and personalized assistance—qualities that many find lacking in human-mediated or socially curated content (Logg et al., 2019). However, these perceptions appear to be shaped less by evaluations of objective neutrality than by information fatigue, emotionally charged online narratives, and a pragmatic desire for relief from the uncertainties associated with social media environments, as highlighted in recent research on public discourse around digital threats (Achuthan et al., 2025). Participants report leveraging chatbots for budgeting advice, homework assistance, and mental-health check-ins, valuing AI’s rapid synthesis of large data volumes (Kasneci et al., 2023). Yet a substantial minority (25.9%) remains skeptical, voicing concerns about hidden biases, data-privacy risks, and the inscrutability of complex models. From a broader theoretical perspective, this pattern aligns with discussions of authenticity and mediated interaction in digital environments (Turkle, 2011), as well as critiques of surveillance-based platform economies that emphasize users’ growing desire for more controllable and less socially manipulative digital tools (Zuboff, 2019).

For policymakers and technologists, these insights point to a dual imperative: first, to expand AI literacy efforts that demystify this study indicate that young people’s relationship with the digital world should be addressed not only through individual preferences and personal coping strategies, but also at the level of social policy. From an educational perspective, systematically integrating digital minimalism and critical media literacy into curricula from secondary school through higher education may support young people in managing screen-time pressures and intensive digital steering in a more balanced way. With regard to digital platforms, increasing transparency about content sources, clarifying the boundary between advertising and news, and strengthening public communication about how recommendation systems operate may help reinforce young people’s trust in digital environments. In the financial domain, combining youth-oriented financial literacy initiatives with simple, accessible, and everyday budgeting tools may contribute to the development of more sustainable spending and saving behaviors. Finally, in the field of artificial intelligence, expanding AI literacy programs that take young people’s actual usage patterns into account, while promoting systems with more understandable decision-making processes, appears crucial both for maintaining trust and for strengthening a culture of responsible use. This emphasis directly reflects participants’ accounts of using AI tools pragmatically as decision-support resources in response to distrust in social media and cognitive overload, rather than as unquestioned sources of authority.

The author is the founder of a youth-focused civil society organization and has been actively involved in both academic and applied work on digitalization, including studies on digital minimalism, the impact of digital technologies on family life, and young people’s financial capabilities. In addition, the author teaches elective university courses on youth development and life planning, which has contributed to a close engagement with young adults’ everyday concerns, values, and coping strategies in digitally saturated environments. These professional experiences have shaped the author’s attentiveness to issues such as digital overload, trust, and self-regulation, while also reinforcing the need for interpretive caution. Accordingly, the study does not advance a normative position on digital technologies, artificial intelligence, or digital minimalism. To limit the influence of prior engagements on the analysis, the research relied on open-ended interview questions and a close grounding of interpretations in participants’ own accounts, allowing themes to emerge inductively rather than being guided by pre-existing assumptions.

## 5 Limitations

This study has several limitations that should be considered. As a qualitative and cross-sectional inquiry, it does not seek to establish causal relationships or to trace changes over time; instead, it captures participants’ experiences and interpretations at a specific moment. In addition, the study does not aim to cover all possible factors or functional distinctions related to digital minimalism, financial discipline, or AI-related practices. Although participants mentioned different types of AI-based tools, such as chatbots, recommender systems, and financial applications, these were examined collectively as decision-support resources, reflecting how they were discussed and experienced in everyday life rather than as separate functional categories. Finally, the focus on a specific group of young adults may limit the broader generalizability of the findings. As this study is grounded in the Turkish context, the findings should be read with an awareness of local media, educational, and economic conditions. While the themes identified echo broader Generation Z experiences, they may take different forms across cultural settings. While participants mentioned different social media platforms, this study does not pursue a platform-specific comparison. A more detailed analysis of platform-based differences is beyond the scope of the present research and remains a direction for future studies. Nevertheless, the study provides nuanced insights into how Generation Z navigates digital trust, self-regulation, and emerging AI practices within a contemporary socio-digital context.

## 6 Conclusion

In sum, this study illuminates Generation Z’s dynamic negotiation of digital engagement, revealing that while pervasive social-media distrust and substantial screen-time demands propel many toward digital minimalism, these same pressures concurrently drive a robust turn to AI a pragmatic and comparatively reliable source of decision support. The present study directly addresses this gap by proposing a qualitative integrative model that positions AI-related trust perceptions as a context-dependent mediating dimension linking digital minimalism and financial discipline under conditions of social media distrust. In doing so, the model offers a novel conceptual lens for understanding how emerging technologies reconfigure self-regulation and economic behavior among digitally saturated youth. Despite broad recognition of financial precarity, systematic budgeting practices lag behind, highlighting the need for more accessible, integrated tools and educational supports. These conclusions should be interpreted in light of the study’s qualitative scope and student-weighted sample, which privilege depth of experience over statistical generalizability. By advancing media-literacy curricula, promoting transparent platform governance, and fostering ethically designed AI solutions, policymakers and educators can help young adults cultivate balanced digital-health and financial well-being—ultimately empowering Generation Z to thrive in an increasingly complex techno-social ecosystem. Future research may extend this integrative model through longitudinal and cross-cultural designs to assess how digital minimalism, financial discipline, and AI-related trust perceptions co-evolve across different socio-economic contexts.

## Data Availability

The datasets presented in this article are not readily available because the dataset generated and analyzed during the current study is not publicly available due to the confidential nature of interview data and the privacy agreements with participants. De-identified excerpts or summary findings may be provided by the corresponding author upon reasonable request and subject to ethical approval. Requests to access the datasets should be directed to busraokten@gmail.com.
