# Supplementary material for: Social media distrust and turn to artificial intelligence among generation Z: a qualitative model linking digital minimalism and financial discipline with social policy recommendations
Source: Front Artif Intell. 2026 Mar 19;9:1684312. doi: 10.3389/frai.2026.1684312 (PMC13043641; doi:10.3389/frai.2026.1684312)
Supplement: Supplementary file 1 [file Data_Sheet_1.docx]

Semi-Structured Interview Questions for the Study:

“Social Media Distrust and the Turn to Artificial Intelligence among Generation Z:

A Qualitative Model Linking Digital Minimalism and Financial Discipline with Social Policy Recommendations”

(Study: Social Media Distrust and Turn to Artificial Intelligence Among Generation Z)**

**Participant Basic Information**

**Your Age / Year of Birth**

**Your Gender***

- Female
- Male

**Education Level***

- High School
- University
- Master’s Degree

**Employment Status**

- Student
- Employed (full-time)
- Employed (part-time)
- Unemployed

**City of Residence***

**Your Monthly Personal Income (if any)***

- No income
- 0–10,000 TL
- 10,001–20,000 TL
- 20,001–40,000 TL
- 40,000–60,000 TL
- Above 60,000 TL

**Your Average Daily Digital Device Usage***

- Less than 1 hour
- 1–3 hours
- 4–6 hours
- 7 hours or more

**Main Digital Platforms You Use**
(e.g., Instagram, TikTok, X, YouTube, LinkedIn)

**Main Artificial Intelligence Applications You Use**
(e.g., ChatGPT, Gemini, Claude, Grok, DeepSeek)

**Section 1: Social Media Trust and Information Evaluation**

**How much do you trust the information you encounter on social media?**
(e.g., Do you think the news or content you see on Instagram, TikTok, X, or YouTube is accurate?)

**Have you ever come across false or misleading information on social media?**
*Follow-up:* What did you do in that situation? Did you seek another source?

**What do you think about the accuracy of information shared on social media?**
*Follow-up:* In your opinion, is it easy or difficult to access accurate information in such environments? Why?

**Section 2: Digital Usage and Minimalism**

**How do you evaluate your daily digital device usage?**
(e.g., How many hours are you in front of a screen? Can you give an example?)

**Do you have specific habits to limit your phone or computer usage?**
(e.g., digital decluttering, disabling notifications, screen-time apps, restricting usage to certain hours)
Why?

**Have you ever tried to reduce your daily digital usage?**
Were you successful?
If not, why?
If yes, what changed afterward?
Did you notice any mental or behavioral differences?

**Section 3: Financial Management and Spending Behavior**

**How competent do you feel in managing your own money?**
(e.g., Do you budget? Track your expenses? Have saving or investment plans?)

**Do you have a system or application for planning your expenses?**
(e.g., monthly budgeting sheets, notebooks, mobile apps, bank app expense trackers)

**How easy or difficult is it for you to control yourself when shopping on digital platforms? Why?**
Do social-media ads or content influence your spending decisions?
Have you ever changed your mind at the last moment and made an unplanned purchase?
Can you give an example?

**When you think about a recent “unnecessary purchase,” how do you feel?**
Can you give an example?

**Section 4: AI Trust and Use of Artificial Intelligence**

**Do AI applications help you in your financial decisions?**
(e.g., asking ChatGPT “How can I manage my money?”, using investment or spending-advice apps)
How often do you use them?

**When you encounter information you do not trust, what do you use first to verify it?**
*Follow-up:* Do you use AI tools such as ChatGPT in these situations?

**Have there been times when your trust in social media decreased?**
If yes, did this increase your tendency to use AI tools?
*Follow-up:* Do you now use ChatGPT or similar tools more frequently?

**Does the information pollution on social media affect your trust in AI?**
(e.g., As social-media chaos increases, do you perceive AI as more reliable?)

**Do you trust the recommendations given by artificial intelligence?**
If yes, what features make AI trustworthy for you?
If no, what concerns or doubts do you have?

**Do you think AI could one day make financial decisions as accurately and reliably as humans? Why?**

**Section 5: Digital Habits, Financial Behaviors, and Broader Reflections**

**Do you think your digital habits influence your spending behaviors? How?**

**Have you ever deliberately distanced yourself from social media or digital apps?**
Did you notice changes in your mental or financial behavior during that period?

**Do you think your digital media experiences influence your decision to trust or distrust AI?**
(e.g., If you frequently encounter false or misleading content on social media, do you consult AI tools first when seeking information?)

**In your opinion, can conscious digital-tool usage help a person become more financially balanced? Why?**

**Do you think digital consumption culture leads to more or less financial discipline?**
Which side do you personally experience more strongly?

**Do you think AI might eventually replace or become more preferred than social media?**
*Follow-up:* Do you think this would be beneficial or potentially harmful?

**Do you think governments and NGOs can do anything to promote AI literacy, social-media awareness, or financial consciousness?**
What would you recommend?

**Is there anything you would like to add regarding digitalization, financial behaviors, or artificial intelligence that we have not discussed today?**
